# Hempseed-based diets supplemented with carbohydrase and butyric acid for broiler chickens

**DOI:** 10.1016/j.psj.2026.107622

**Published:** 2026-08-15

**Authors:** Alireza Shahtalab, Farid Shariatmadari, Mohammad Amir Karimi Torshizi, Hamed Ahmadi

**Affiliations:** Department of Poultry Science, Faculty of Agriculture, Tarbiat Modares University, Tehran, Iran

**Keywords:** Hempseed, Butyric acid, Carbohydrase, Broiler

## Abstract

The aim of this study was to evaluate the impacts of hempseed-based diets supplemented with butyric acid and carbohydrase enzyme blend (xylanase + β-glucanase) on growth performance, nutrient digestibility, intestinal morphology, microbial population, and blood biochemistry of broiler chickens. A total of 480 one-day-old male broiler chicks were assigned randomly to five treatments (8 replicates each; 12 birds per pen) described as follows: a corn–soybean control (C), a 16 % hempseed (H), and hempseed + carbohydrase blend enzyme (HE), hempseed + butyric acid (HBA), and hempseed + carbohydrase + butyric acid (HEBA). Sixteen percent of hempseed as a replacement for soybean and additives was administered from days 8 to 28. Hempseed diets, particularly HE and HEBA, demonstrated higher body weight gain and lower feed conversion ratio compared with the control (p < 0.05). Birds fed HEBA diet had the lowest counts of total aerobic bacteria, E. coli, and Lactobacillus, and had significant difference compared control (p < 0.05), while showed higher calcium, and phosphorus levels in their serum (p < 0.05). Hempseed-based diets enhanced intestinal morphology, with greater villus width and villus surface area in the ileum and HBA treatment improved crypt depth and villus height/crypt depth ratio in the jejunum. Apparent ileal digestibility of dry matter, gross energy, crude protein, and ether extract increased in H and HE. Overall, replacing 16 % hempseed with carbohydrase blend and butyric acid can improve nutrient utilization, intestinal health, and immune status without adverse metabolic effects, confirming hempseed’s potential as a sustainable and functional ingredient for broiler nutrition.

## Introduction

In recent years, there has been growing interest in incorporating alternative feed ingredients into poultry diets to reduce dependence on traditional resources like soybean meal and corn. This shift is driven by rising feed costs, the need for sustainable production, and the potential to utilize underexplored nutrient sources.

Hemp (Cannabis sativa) has emerged as a promising candidate due to its high-quality protein, favorable fatty acid composition, and bioactive compounds. Hempseed contains approximately 25 % crude protein, a highly digestible amino acid profile, functional oils rich in **polyunsaturated fatty acids (PUFAs)**, and high concentrations of minerals, vitamins, and phenolic compounds, making it a potentially valuable poultry feed ingredient (Shariatmadari, 2023). It also provides natural antioxidants (e.g., tocopherols) and antimicrobial agents (notably cannabinoids) that may support poultry health and immunity.

However, the high fiber content and presence of antinutritional factors in hemp can impair nutrient utilization, reduce nutrient bioavailability, and negatively influence growth performance, thus limiting its direct inclusion in poultry diets (Montero et al., 2023). The majority of the fiber fraction in hempseed is concentrated in the seed coat, which represents the primary structural barrier to nutrient accessibility. Hempseed fiber consists predominantly of insoluble fiber, with an approximate soluble-to-insoluble fiber ratio of 20:80. Hempseed fiber is rich in structural NSPs such as cellulose, arabinoxylans, β-glucans, and pectic substances, along with lignin-associated cell wall material. Because poultry lack sufficient endogenous carbohydrases to hydrolyze these polysaccharides, they remain largely indigestible and may encapsulate intracellular nutrients, thereby limiting enzyme access and reducing nutrient utilization. Both insoluble and soluble NSP fractions can impair digestion, the former by forming a physical barrier and the latter by modifying digesta properties and increasing viscosity (Tejeda and Kim, 2021).

To overcome these challenges, nutritional strategies such as dietary additives have been explored. Multienzyme preparations (including cellulase, glucanase, xylanase, phytase, and protease) break down non-starch polysaccharides and other complex plant components, improving nutrient digestibility and energy availability in high-fiber diets (Yuan et al., 2017). Exogenous carbohydrases, particularly xylanase and β-glucanase, are effective feed additives for enhancing the utilization of fibrous ingredients in poultry diets. Xylanase hydrolyzes arabinoxylan backbones, while β-glucanase degrades β-glucan polymers, thereby disrupting plant cell-wall integrity and facilitating the release of encapsulated nutrients (Bedford et al., 2024). Butyric acid and exogenous enzymes are well-established feed additives in broiler nutrition because they improve gut health, nutrient digestibility, and feed efficiency. Butyrate supports intestinal epithelial integrity, modulates microbiota, and enhances immune function, whereas exogenous enzymes increase the utilization of fibrous and anti-nutritive components in plant-based diets. Short-chain fatty acids like Butyric acid, through diminishing intestinal pH, suppress bacterial pathogenesis and improve intestinal morphology, can ameliorate gut health. Butyric acid specifically boosts intestinal epithelial cell integrity, develops villus height-to-crypt depth ratio, and improves nutrient absorption efficiency by stabilizing gut microbiota and supporting beneficial bacteria such as Lactobacillus (Dibner and Buttin, 2002). When applied together, organic acid and multienzymes may exert combined effects, further enhance feed efficiency and supporting broiler performance (Yaseen et al., 2024).

Several studies have evaluated hempseed inclusion in poultry diets; the findings remain inconsistent. For instance, Khan et al. (2010) reported that the seed powder of hemp, when added to the feed at the rate of 20 %, has a positive effect on carcass quality of broiler chicks. It will also decrease the market age and mortality rate which subsequently decrease the productive cost. Konca et al. (2014) revealed that 10 % HS supplementation to the diet increased carcass and heart weight but decreased liver and intestinal weight; However, gizzard weight was not affected in the fattening period of quail. Other research suggests that hemp can enrich poultry products with omega-3 fatty acids and bioactive compounds, but its high fiber and ash contents often limit energy utilization (House et al., 2010; Yalcin et al., 2018). Mahmoodtabar et al. (2026) performed the treatment of Raw and heated hemp with or without a carbohydrase blend (glucanase and xylanase) and concluded that raw hempseed can be promising and beneficial in broiler feeding, improving performance and feed intake. Recent approaches, such as heating hempseed cake with phytase or combining hemp with flaxseed, have shown partial improvements in digestibility and metabolism. Likewise, organic acids have consistently demonstrated benefits for gut morphology, immunity, and growth performance (Darmawan and Ozturk, 2025). However, most of these studies focus on hemp or feed additives individually, and the combined effects of organic acids and multienzymes in hemp-based diets remain poorly understood.

Therefore, this study was conducted to evaluate the effects of hempseed-based diets supplemented with butyric acid and carbohydrase blend on productive, blood, and gastrointestinal traits of broiler chickens.

## Materials and methods

The animal care of the present experiment was approved by the Animal Care and Use Committee at Tarbiat Modares University (IR. MODARES. REC.1400.032).

### Birds, housing and management

A total of 480 one-day-old Ross 308 broiler chicks were obtained from an industrial hatchery and randomly assigned to five treatments and eight replicates with 12 chicks each. Each pen of replicate (2.0×1.0×0.8m) was equipped with nipple drinkers, one trough feeder, and 5 cm of fresh wood shavings as litter. In the first week of the rearing period, the room temperature was maintained at 33°C, and then, to reach 22°C, it was reduced by 2.5°C weekly. Lighting was continuous for the first two days, followed by a gradual increase in the dark period to reach 7 h by day 7, which was maintained thereafter. Throughout the experiment, feed and water were available ad libitum.

### Experimental design and treatments

The experiment was conducted as a completely randomized design with five dietary treatments that were as follows: a control diet based on **corn-soybean based (C); a diet containing 16** % **hempseed as a replacement for soybean meal (H); 16** % **hempseed supplemented with enzymes (HE); 16** % **Hempseed supplemented with butyric acid (HBA); and 16** % **Hempseed supplemented with both enzymes and butyric acid (HEBA)**. The diet was consistent for all the birds until day 7, and the experimental treatments were applied from day 8 until the end (day 28). The percentage of hempseed replacement was chosen based on a previous article (Rasool, 2018). The ingredients and nutrients contents of the experimental treatments were presented in Table 1. The experimental diets were analysed for **crude protein (CP), neutral detergent fibre (NDF), acid detergent fibre (ADF)**, and **gross energy (GE)**. Crude protein was determined according to the official methods of AOAO (2000), NDF and ADF were analysed according to Van Soest et al. (1991) and GE was measured using an adiabatic bomb calorimeter (Gallenkamp Autobomb, UK). Nutrient composition of hempseed illustrated in Table 2.

**Table 1:** Composition and nutrient contents of experimental diets (as-fed basis).

Ingredients (g kg^−1^)	1-7 day	8-28 day				
		C	H	HE	HBA	HEBA
Corn	529	523.05	476.02	475.92	475.02	474.92
Soybean meal 44	400	393.11	307.13	307.13	307.13	307.13
Soybean oil	21.5	42.47	14.21	14.21	14.21	14.21
Calcium carbonate	16.1	14.00	14.76	14.76	14.76	14.76
Salt	4	3.83	3.88	3.88	3.88	3.88
Hempseed	-	-	160	160	160	160
Vitamin/mineral premix^a^	5	5.00	5.00	5.00	5.00	5.00
Monocalcium phosphate	17	14.37	12.54	12.54	12.54	12.54
Lysine	2.5	0.91	2.03	2.03	2.03	2.03
Threonine	1.2	0.33	0.96	0.96	0.96	0.96
Methionine	3.7	2.93	3.48	3.48	3.48	3.48
Butyric acid	-	-	-	-	1.00	1.00
Carbohydrase	-	-	-	0.10	-	0.10
Total	1000	1000	1000	1000	1000	1000
ME (Kcal/kg)	2890	3030	3030	3030	3030	3030
CP %	22.11	21.5	21.5	21.5	21.5	21.5
Lys %	1.38	1.24	1.24	1.24	1.24	1.24
Met + Cys %	1.03	0.95	0.95	0.95	0.95	0.95
Thr %	0.94	0.84	0.84	0.84	0.84	0.84
Available P %	0.50	0.44	0.44	0.44	0.44	0.44
Ca %	1.00	0.87	0.87	0.87	0.87	0.87
Na %	0.17	0.16	0.16	0.16	0.16	0.16
Ca/P availability %	2.00	2.00	2.00	2.00	2.00	2.00
Nutrient composition						
Crude protein (%)		21.60	21.35	21.40	21.53	21.30
Gross energy (Kcal/kg)		3492	3615	3638	3643	3606
Neutral detergent fiber (%)		31.82	34.02	32.84	33.67	33.79
Acid detergent fiber (%)		18.09	22.44	21.88	22.28	21.92

a Each kg of vitamin and mineral premix contained: Vitamin A 4000000 IU, vitamin E 26000 IU, vitamin D3 1800000 IU, vitamin K 1200 mg, vitamin B1 1000 mg, vitamin B2 2600 mg, Niacin 5400 mg, Pantothenic Acid 7500 mg, vitamin B6 1280 mg, Folic acid 760 mg, Biotin 72 mg, vitamin B12 6.8 mg, choline chloride 320000 mg and antioxidant 1000 mg, Fe, 8000 mg, Mn, 48000 mg, Cu, 6400 mg, I, 500 mg, Zn, 44000 mg, Se, 120 mg. Carbohydrase: endo-1,3(4)-β-glucanase and endo-1,4-β-xylanase. Titanium dioxide (TiO₂) was included at 5 g/kg in all experimental diets as an indigestible marker for determination of apparent ileal digestibility.

**Table 2:** Nutrient composition of Hempseed.

Hempseed composition	
Dry matter (%)	94.62
Gross energy (Kcal/kg)	5925
Crude protein (%)	24.7
Ether extract (%)	30.5
Ash (%)	5.37
Crude fiber (%)	29.6
Neutral detergent fiber (%)	32.4
Acid detergent fiber (%)	22.1
Total Lysine (%)	1.02
Total Methionine (%)	0.43
Total Threonine (%)	0.62

Two NSPase enzymes, Econase XT (endo-1,4-β-xylanase, minimum activity of 4,000,000 BXU/g) and Econase GT 200 (endo-1,3(4)-β-glucanase, minimum activity of 200,000 BU/g), were sourced from AB Vista, Netherlands. Xylanase activity was determined by measuring the release of reducing sugars from 1 % birchwood xylan in 0.1 M sodium acetate buffer (pH 5.5) at 37°C. After a 30-minute incubation with the enzyme, **dinitrosalicylic acid (DNS)** reagent was added and the mixture was boiled for 5 min. Absorbance was measured at 540 nm using a microplate reader. One **unit (U)** of xylanase activity was defined as the amount of enzyme that releases 1 µmol of reducing sugar per minute. Enzyme activity was calculated based on a xylose standard curve and expressed in U/g. All assays were performed in triplicate, and results with a **relative standard deviation (RSD)** < 5 % were accepted (Dhaver et al., 2022). Xylanase activity measurements yielded values of 4,520,000, 4,380,000, and 4,495,000 U/g in three independent replicates. The mean activity was calculated as 4,465,000 U/g, with RSD of 1.59 %, indicating high precision and reproducibility of the assay. The activity of a commercial β-glucanase preparation was measured using the DNS assay with 1.5 % barley β-glucan as the substrate. The enzyme was incubated at 40°C for 20 min in 100 mM acetate buffer (pH 5.0), and the reducing sugars released were quantified by measuring absorbance at 530 nm. The β-glucanase activity for the three replicates was found to be 223,000 U/g, 236,500 U/g, and 218,000 U/g, with a mean of 225,500 U/g and RSD of 4.08 %, indicating high precision and reproducibility of the assay. The organic acid treatment in this study utilized Butyrin Ultra, a microencapsulated butyric acid glyceride developed for poultry diets produced by Adisseo company.

### Growth performance

Individual **body weight (BW)** was recorded upon chick placement (40.0 ± 0.2 g), on day 7 immediately before the experimental diets were introduced, and again on day 28. **Body weight gain (BWG)** corresponds to the period during which the experimental diets were applied, and was calculated from day 8 to 28. **Feed intake (FI)** was determined on a pen basis by recording the amount of feed offered and subtracting the feed residuals at the end of the trial. **Feed conversion ratio (FCR)** was calculated as the ratio of FI to BWG for each replicate pen. All growth performance data were expressed on a pen basis, with cage serving as the experimental unit.

### Ileal microbial population

At day 28, two birds per cage were selected (BW close to the group average), slaughtered, and sampled for intestinal bacterial populations. The ileum, defined as the segment from Meckel’s diverticulum to 5 cm anterior to the ileocecal junction, was excised and cut open to collect approximately 1 g of mixed and homogenized digesta. The droplet count method was utilized in a saline solution to distinguish the **Colony Forming Units (CFU)**. Each sample of ileal contents were homogenized, and then 1 gram of each sample was collected and transferred into 9 ml sterile saline solution to prepare serial dilutions. Plate Count Agar (Merck, Darmstadt, Germany), MacConkey Agar (Himedia Laboratories, Mumbai, India), and MRS Agar (Merck, Darmstadt, Germany) were used for enumeration of total aerobic bacteria, E. coli, and Lactobacillus, respectively (Jabbar et al., 2024). All plates were incubated aerobically at 37°C for 24 h before colonies were counted.

### Intestinal morphology assessment

To evaluate intestinal morphology, 2 cm segments of the duodenum, ileum, and jejunum from two birds per pen were collected. These samples were precisely preserved in a 10 % buffered formalin saline solution to ensure the integrity of the tissue. The dehydration process was applied by immersing the segments in a series of alcohols with increasing concentrations, from 70 % to absolute, followed by infiltration with xylene and embedding in paraffin wax. Using a light microscope (Standard 20, Carl Zeiss, Göttingen, Germany) equipped with imaging software (Dino-Capture 2.0, Taiwan), **villus surface area (VSA), villus height (VH), crypt depth (CD), villus width (VW)**, and **goblet cell count (GN)** were measured (six well-oriented villi per each sample). The **villus height-to-crypt depth (VH/CD)** ratio was subsequently calculated for each sample.

### Apparent ileal digestibility

At the end of the rearing period, **ileal apparent digestibility (IAD)** was determined by choosing 2 birds per replicate (BW close to the group average) and the ileal digesta after euthanized by cervical dislocation were collected. The ileal contents were gently flushed with distilled water into labeled containers and pooled by replicate. Samples were immediately frozen at –20°C, and later oven-dried at 55°C. **Dry matter (DM), organic matter (OM),** CP**, crude fiber (CF), and ether extract (EE)** of ileal content were analyzed according to AOAC (2000). Neutral detergent fiber and acid detergent fiber analysis were performed as described by Van Soest et al. (1991). The GE values of the ileal content were measured using an adiabatic calorimetric bomb (Gallenkamp Autobomb, UK). All experimental diets were supplemented with titanium dioxide (TiO₂) at 5 g/kg as an indigestible marker. Titanium dioxide concentration in both diets and ileal digesta was determined according to the method of (Short et al., 1996). IAD coefficients of nutrients were calculated using the following marker ratio formula:

$$Ileal\;apparent\;digestability\;\%=100\;\times \;\left(1-\frac{Nutrient_{digesta}/Marker_{digesta}}{Nutrient_{diet}/Marker_{diet}}\;\right)$$

### Blood sampling and biochemical parameters

Two birds (per replicate) were selected for sampling, and blood samples were obtained from the brachial (wing) vein using a 21-gauge needle. The collected blood was transferred into clot-activator tube. Serum was separated by centrifugation (1500 x g for 20 min) and kept at −20°C until subsequent analysis.

**Total protein (TP), albumin (Alb), globulin (Glo), glucose (Glu), cholesterol (Chol), high-density lipoprotein (HDL), low-density lipoprotein (LDL), triglycerides (TG), calcium (Ca), and phosphorus (P)** of serum were determined by commercial diagnostic kits (Pars Azmoon Co., Tehran, Iran) using an auto-analyzer.

### Statistical analysis

The experiment was conducted using a **completely randomized design (CRD)**. The replicate cage served as the experimental unit for all statistical analyses. Prior to analysis, the residuals were examined for potential outliers, and no observations were excluded. The normality of the residuals was assessed using the Shapiro–Wilk test. Data were analyzed by the GLM procedure of SAS software (SAS Institute, 2013). A significance level of P < 0.05 was applied and significant differences were identified using Tukey's test.

## Results and discussion

### Growth performance

Growth performance variables were significantly affected by dietary treatments at 28 days of age (Table 3). Feed intake was lower in the HEBA group than in the HBA group (P < 0.05), whereas the remaining treatments did not differ significantly. Birds fed the H and HE diets exhibited greater final body weight than the control group (P < 0.05). Body weight gain was higher in the HE group than in the control group (P < 0.05), while the H, HBA, and HEBA groups showed intermediate values that did not differ significantly from either treatment. Feed conversion ratio was improved in the H, HE, and HEBA groups compared with the control group (P < 0.05), whereas the HBA group did not differ from either treatment. Overall livability was 97.3 %, with no visible signs of illness or disease throughout the experimental period.

**Table 3:** Growth performance of treatments at 28 days of age.

Treatments*	Feed intake (g)	Body weight (g)		Body weight gain (g)	Feed conversion ratio
	8 – 28 Day	7 Day	28 Day	8 – 28 Day	8 – 28 Day
C	1366.6^a^^,^^b^	164.79^a^	1171.8^b^	1002.4^b^	1.37^a^
H	1391.2^a^^,^^b^	167.29^a^	1271.1^a^	1085.1^a^^,^^b^	1.28^b^
HE	1370.8^a^^,^^b^	163.22^a^^,^^b^	1259.5^a^	1086.1^a^	1.26^b^
HBA	1399.6^a^	157.18^b^	1214.0^a^^,^^b^	1068.0^a^^,^^b^	1.31^a^^,^^b^
HEBA	1312.4^b^	162.39^a^^,^^b^	1203.9^a^^,^^b^	1041.5^a^^,^^b^	1.26^b^
SEM	19.85	2.18	21.34	20.34	0.027
*P-value*	0.033	0.034	0.013	0.031	0.044

a-c Means in the same column with different superscripts are significantly different (p < 0.05).

⁎ Eight replicate cages per treatment. SEM: standard error of the mean. C: Control, H: 16 % hempseed, HE: 16 % hempseed with enzyme, HBA: 16 % hempseed with butyric acid, HEBA: 16 % hempseed with enzyme and butyric acid.

According to the results, the increase in FI in the hempseed treatment, which aligns with previous research, is likely due to the appetite-stimulating effect of **Δ⁹-tetrahydrocannabinol (THC)** (Mahmoudi et al., 2015). The appetite-stimulating potential of hempseed may also be related, in part, to its naturally occurring cannabinoid fraction, including trace amounts of THC and other phytocannabinoids. THC acts as a partial agonist of **cannabinoid receptor type 1 (CB1)**, a key component of the endocannabinoid system involved in the central regulation of appetite, energy balance, and feeding behavior. Activation of CB1 receptors has been shown to stimulate feed intake through modulation of hypothalamic orexigenic signaling and reward pathways, whereas pharmacological blockade of these receptors suppresses appetite (Alizadeh et al., 2015; Hassan et al., 2023). In this study, dietary inclusion of 16 % hempseed enhanced BW and BWG compared with the control diet, while FCR was also improved in hempseed-based diets, particularly when combined with carbohydrase supplementation. These findings agree with earlier reports indicating that hempseed can partially replace soybean meal without compromising growth, and in some cases may even improve BWG and FCR when properly processed and formulated (Khan et al., 2010; Sana et al., 2024; Tufarelli et al., 2023).

The superior performance (refer to FCR) of HE and HEBA compared with H alone suggests that carbohydrase supplementation and butyrate act on at least partially overlapping physiological pathways rather than independent ones. Xylanase and β-glucanase degrade the hempseed cell wall matrix, releasing entrapped nutrients and generating fermentable oligosaccharide fragments; in broilers fed enzyme-supplemented fibrous diets, this NSP degradation has been directly linked to increased **short-chain fatty acid (SCFA)** production in the distal gut (Kouzounis et al., 2021). Because dietary butyrate supplementation and enzyme-derived endogenous SCFA production converge on the same epithelial signaling pathways (Salvi and Cowles, 2021). The combination of carbohydrase and butyric acid in HEBA may reinforce, rather than simply add to, a shared mechanistic axis. This provides a physiological basis for the numerically or statistically superior BWG and FCR observed in enzyme- and butyrate-supplemented groups relative to hempseed alone, beyond the appetite-stimulating effect of THC discussed above.

Carbohydrase supplementation contributed to improved efficiency, consistent with evidence that enzymes enhance nutrient digestibility and support weight gain without altering FI (Attia et al., 2022; Yi et al., 2024). Butyric acid also showed benefits, as reflected in the HBA group’s performance, though the combination of both in HEBA yielded intermediate results, in line with previous report (Paul et al., 2007). Another experience on performance with hempseed diets supplemented by additives aligns with broiler trials showing that dietary butyric acid improves BWG and FCR and helps maintain carcass quality under challenge (Makowski et al., 2022). Overall, the results indicate that hempseed can be safely included at 16 % of the diet, and its value can be maximized with multienzyme or butyric acid supplementation to sustain growth performance in broilers.

Nutrient digestibility amplification was another key factor contributing to the better FCR. Multienzymes improve the breakdown of NSPs and cell wall components, releasing nutrients for absorption (Attia et al., 2022). Our improvements in BWG and FCR with carbohydrase supplementation align with studies showing that xylanase + β-glucanase enhance growth performance in broilers by breaking down blend carbohydrates in the diet, thereby improving nutrient availability (Daneshmand et al., 2023). A newer monocomponent xylanase also improved performance and feed efficiency while modulating the intestinal environment, supporting our findings (Vasanthakumari et al., 2023).

Growth enhancement caused by organic acid can be attributed to lowering intestinal pH, reducing pathogenic bacteria, and stimulating digestive enzyme activity (Ma et al., 2021). Butyrate acidifies the gastrointestinal tract and supports intestinal maturation, which can enhance nutrient utilization and growth. Studies using butyric acid glycerides similarly report better live performance and favorable carcass traits in broilers (Antongiovanni et al., 2007). Reviews conclude that butyric acid (including protected forms) improves performance by lowering digesta pH, reducing pathogenic bacteria, and supporting gut integrity (Abd El-Ghany, 2024).

Interestingly, feed intake was reduced in the HEBA group, which may indicate improved nutrient availability per unit of feed and reduce the need for more feed to achieve growth targets. On the other hand, appetite regulation following a change in intestinal fermentation patterns may cause this issue. According to results, dietary hempseed significantly improved BWG and FCR compared to the control, suggesting that it can replace soybean meal without negative impact on performance, that probably due to hempseed’s high lipid content, which increases the energy density of the diet, thus reducing the amount of feed needed for growth. Enhanced fat digestibility and energy availability owing to the existence of PUFAs in hempseed could explain the better FCR in hempseed-fed broilers (Sana et al., 2024).

### Ileal microbial populations

Dietary treatments significantly affected ileal microbial populations at 28 days of age (Table 4). Total aerobic bacterial counts were lower in the HEBA group than in the C, H, and HE groups (P ≤ 0.05), whereas the HBA group showed intermediate values. Lactobacillus counts were highest in the C and H groups and lowest in the HEBA group (P < 0.05), while the HE and HBA groups exhibited intermediate values. Escherichia coli counts were highest in the HBA group and lowest in the HEBA group (P < 0.05). The H group showed lower E. coli counts than the control group, whereas the HE group exhibited intermediate values that did not differ significantly from either the control or HBA groups.

**Table 4:** Ileal microbial populations of treatments at 28 days of age (log_10_CFU/g).

Treatments*	Total aerobic bacteria	Lactobacillus	E. coli
C	8.14 a	8.11 a	4.73 b
H	8.09 a	8.13 a	3.86 c
HE	8.12 a	8.06 b	5.09 a^,^^b^
HBA	8.01 a^,^^b^	8.09 a^,^^b^	5.66 a
HEBA	7.85 b	7.81 c	2.83 ^d^
SEM	0.064	0.009	0.138
*P-value*	0.050	<0.001	<0.001

a-c Means in the same column with different superscripts are significantly different (p < 0.05).

⁎ Eight replicate cages per treatment. SEM: standard error of the mean. C: Control, H: 16 % hempseed, HE: 16 % hempseed with enzyme, HBA: 16 % hempseed with butyric acid, HEBA: 16 % hempseed with enzyme and butyric acid.

The ileal microbial population was significantly influenced by hempseed diets, especially when enrichment with carbohydrase blend and butyric acid in treatment HEBA. The reduction in total aerobic bacteria in HEBA group indicates that carbohydrase and butyric acid overall suppressed bacterial load. Organic acids diminished intestinal pH and exerted direct antimicrobial effects, while multienzymes leading to fewer bacteria were due to improvement in digestibility and a reduction in undigested nutrients available for microbial growth (Anjum and Chaudhry, 2010).

Despite nutrient utilization improving, Lactobacillus spp. were reduced in the HE and HEBA groups. Aligned outcomes were reported by Yaseen et al. (2024); a reduction in these species of bacteria may be due to competitive shifts by limiting Lactobacillus populations and fewer substrates for fermentation. Notably, Lactobacillus was maintained at control levels in the H group, suggesting hempseed itself supports beneficial bacteria, likely due to its soluble fiber fraction. Previous report based on combined xylanase+β-glucanase program modulated jejunal microbiota composition alongside performance improvements, paralleling our enzyme effects (Daneshmand et al., 2023).

E. coli opposite responses determined that the highest and lowest counts occurred in HBA and HEBA, respectively. Probably, the decrease of Lactobacillus leads to the reduction of competition, and then the growth of E. coli is allowed. Conversely, the marked suppression of E. coli in HEBA indicates a strong synergistic effect of butyric acid and carbohydrase blend in controlling pathogens by lowering pH and limiting nutrient flow to the distal gut. This agrees with Attia et al. (2022), who showed additive combinations can effectively reduce harmful bacteria in broilers.

Beyond pH-mediated antimicrobial action, butyrate may have limited pathogen colonization in the HEBA group through reinforcement of the epithelial barrier itself. Butyrate has been shown to upregulate tight junction proteins (e.g., claudins) and preserve barrier integrity via Akt/mTOR-mediated protein synthesis (Wang and Xiong, 2019), which would restrict the paracellular route pathogenic bacteria such as E. coli exploit to colonize and translocate across the mucosa. In parallel, carbohydrase-mediated degradation of hempseed NSPs likely reduced the pool of undigested fermentable substrate reaching the distal ileum, limiting the nutrient availability that opportunistic E. coli populations depend on for proliferation. The combined action of these two mechanisms (direct antimicrobial/barrier reinforcement from butyrate and substrate restriction from enzymatic NSP breakdown) offers a more complete explanation for why E. coli suppression was greatest in HEBA than either mechanism alone would predict.

Our marked reduction in E. coli (HEOA) is consistent with butyrate trials showing lower coliform/E. coli counts and pathogen suppression in broilers given tributyrin/sodium butyrate (Hu et al., 2021). Protected (encapsulated) forms of butyrate tend to be more effective distally in the gut and have repeatedly shown reductions in E. coli, and C. perfringens with little to no depression of Lactobacillus, supporting our interpretation of a healthier microbial balance under HEOA (Makowski et al., 2022). When organic acids are combined with enzymes, broiler studies measuring cecal bacteria also report pathogen reductions and favorable microbiota shifts—consistent with our HEOA synergy (Chowdhury et al., 2021).

### Intestinal morphology

The effects of dietary treatments on morphometric characteristics of the duodenum, jejunum, and ileum are illustrated in Table 5. In the duodenum, none of the measured parameters were significantly affected by diet (P > 0.05). However, broilers fed the HEBA diet exhibited numerically greater VH, VSA, and VH/CD ratio.

**Table 5:** Morphometric characteristics of the duodenum, jejunum, and ileum of treatments at 28 days of age.

Treatments*	VH (µm)	CD (µm)	VW (µm)	GN (100µm)	VH/CD	VSA (mm^2^)
	Duodenum					
C	1607.0	288.6	89.5	11.5	5.69	0.449
H	1726.4	264.4	90.3	11.1	6.58	0.486
HE	1597.7	253.0	110.3	12.6	6.66	0.562
HBA	1657.3	280.7	97.3	9.5	5.97	0.506
HEBA	1763.2	234.6	106.6	12.1	7.69	0.589
SEM	68.55	17.19	7.49	0.924	0.553	0.046
*P-value*	0.367	0.210	0.213	0.177	0.133	0.219
	Jejunum					
C	903.5	312.7^a^	91.3	12.8	2.90^c^	0.257
H	980.3	246.6^b^^,^^c^	88.2	12.5	3.96^a^^,^^b^	0.272
HE	840.9	261.0^a^^,^^b^^,^^c^	89.8	13.6	3.22^b^^,^^c^	0.236
HBA	965.6	207.4^c^	90.5	13.0	4.77^a^	0.273
HEBA	889.4	268.8^a^^,^^b^	102.3	13.5	3.31^b^^,^^c^	0.284
SEM	58.92	14.64	5.92	1.15	0.224	0.022
*P-value*	0.455	<0.001	0.474	0.950	<0.001	0.609
	Ileum					
C	515.1	202.0	58.2^b^	14.6	2.64	0.094^b^
H	518.9	178.8	83.3^a^	16.1	2.95	0.136^a^^,^^b^
HE	592.4	217.1	82.3^a^	14.8	2.75	0.153^a^
HBA	580.7	179.9	85.6^a^	11.0	3.27	0.155^a^
HEBA	497.1	180.7	89.9^a^	12.5	2.84	0.140^a^
SEM	32.73	15.69	4.96	1.32	0.227	0.011
*P-value*	0.180	0.337	0.001	0.075	0.353	0.004

a-c Means in the same column with different superscripts are significantly different (p < 0.05).

⁎ Eight replicate cages per treatment. SEM: standard error of the mean. C: Control, H: 16 % hempseed, HE: 16 % hempseed with enzyme, HBA: 16 % hempseed with butyric acid, HEBA: 16 % hempseed with enzyme and butyric acid. VH: villus height, CD: crypt depth, VW: villus width, GN: goblet cell count, VH/CD: villus height/crypt depth, VSA: villus surface area.

In the jejunum, CD and VH/CD ratio were markedly influenced by diet. The HBA group exhibited the lowest CD and consequently the highest VH/CD ratio, while the control and HE groups had lower ratios (P < 0.05). Other jejunal parameters did not differ among treatments.

In the ileum, treatments significantly affected VW and VSA (P < 0.05). Birds fed hempseed-containing diets (H, HE, HBA, HEBA) showed wider villi and increased surface area compared to the control group. Oppositely, no significant effects were observed on VH, CD, VH/CD ratio, or GN.

Gut morphology is essential for evaluating digestive efficiency, as it affects nutrient absorption (Stillhart et al., 2020). Increased villus height maximizes absorptive surface area, while reduced crypt depth indicates more efficient cell turnover. Although some of jejunal traits were unaffected after administering treatments, HBA showed reduced CD and an increased VH/CD ratio. This issue implies lower epithelial turnover with more mature villi. Nutrient absorption efficiency enhancement and stabilizing intestinal pH and microbial populations can be suggested by this pattern, which reflects the ability of organic acids to do so (El-Ghany, 2024). A rise in VH/CD ratio in HBA treatment in the jejunum agrees with the previous report that applied tributyrin in broiler feed (Hu et al., 2021). Although xylanase in fiber-rich diets led to an elevation in VH and VH/CD, but inconsistent with our results when we used a blend of xylanase and glucanase (Karimipour et al., 2025).

In the ileum, absorptive surface area expansion can probably be considered as a compensatory response to the fibrous portion of hempseed and resulted in greater VW and VSA in the hempseed-fed groups compared control. Importantly, VH and GN remained unchanged, meaning that the increased surface area came mainly from villus widening rather than elongation, and mucin-secreting capacity was not impaired. This adaptation is particularly valuable in high-fiber diets, where maximizing absorptive area helps maintain nutrient assimilation (Incharoen et al., 2025). Effects of multienzyme and organic acid supplementation on intestinal morphology in similar experiences on high-fiber diets have been reported (Attia et al., 2022; Yaseen et al., 2024). Butyric acid glycerides-related study on the intestinal morphometric parameters of broilers agrees with our observations of VW and VSA in the ileum (Antongiovanni et al., 2007). Methodologically, our calculation of VSA follows established poultry histomorphometry approaches used in hempseed and butyrate literature (Tufarelli et al., 2023).

### Apparent ileal digestibility

Dietary treatments significantly affected the apparent ileal digestibility of DM, CP, EE, NDF, and GE, whereas OM and ADF digestibility were not influenced (Table 6). Dry matter digestibility was higher in the control group than in the HBA group (P < 0.05), while the remaining treatments did not differ significantly. Crude protein digestibility was greater in the H, HE, and HEBA groups than in the control group (P < 0.05), whereas the HBA group showed intermediate values. Ether extract digestibility was higher in the H group than in the control and HBA groups (P < 0.05). Neutral detergent fiber digestibility was lower in the HEBA group than in the C, H, and HE groups (P < 0.05), whereas the HBA group exhibited intermediate values. Gross energy digestibility was greater in the H, HE, and HEBA groups than in the control group (P < 0.05), while the HBA group did not differ significantly from the other treatments.

**Table 6:** Apparent ileal digestibility of treatments at 28 days of age.

Treatments*	DM (%)	OM (%)	CP (%)	EE (%)	ADF (%)	NDF (%)	GE (kcal/kg)
C	89.3^a^	45.5	48.8^c^	72.9^b^^,^^c^	37.3	57.1^a^	40.6^b^
H	88.7^a^^,^^b^	49.9	58.4^a^	86.6^a^	35.2	51.6^a^^,^^b^	50.1^a^
HE	88.5^a^^,^^b^	46.0	56.5^a^^,^^b^	82.8^a^^,^^b^	35.2	53.2^a^	47.0^a^
HBA	87.5^b^	43.4	51.5^b^^,^^c^	67.8^c^	31.7	45.4^b^^,^^c^	45.4^a^^,^^b^
HEBA	88.2^a^^,^^b^	46.5	60.1^a^	77.5^a^^,^^b^^,^^c^	32.1	43.1^c^	47.1^a^
SEM	0.281	1.297	1.341	2.492	1.633	1.421	1.389
*P-value*	0.011	0.056	<0.001	0.002	0.163	<0.001	0.008

a-c Means in the same column with different superscripts are significantly different (p < 0.05).

⁎ Eight replicate cages per treatment. SEM: standard error of the mean. C: Control, H: 16 % hempseed, HE: 16 % hempseed with enzyme, HBA: 16 % hempseed with butyric acid, HEBA: 16 % hempseed with enzyme and butyric acid. DM: Dry matter, OM: Organic matter, CP: Crude protein, EE: Ether extract, ADF: Acid detergent fiber, NDF: Neutral detergent fiber, GE: Gross energy.

Hempseed diets influenced various aspects of nutrient digestibility. The birds fed hempseed lonely had higher CP, EE, and GE compared to the control, as previously reported, which supports our observations (Khan et al., 2010; Sana et al., 2024; Tufarelli et al., 2023). Enhanced EE and GE are most likely due to the high lipid content of hempseed and the enzyme-mediated release of entrapped nutrients. Dry matter decreased in HBA and HEBA groups, in contrast, suggesting that butyric acid, particularly when combined with carbohydrase blend, may have interacted with dietary fiber or altered gut fermentation, slightly diminishing DM utilization (Yaseen et al., 2024). A plausible explanation for the reduced NDF digestibility in HBA and HEBA is that butyrate-driven acidification of the gastrointestinal tract, while suppressing pathogenic and total aerobic populations, may also have reduced the abundance or activity of fiber-fermenting bacteria responsible for degrading residual structural NSPs at the ileal level. Organic acid supplementation has been shown to lower gut pH and reduce microbial populations capable of fermenting digesta, which can paradoxically reduce fiber-related digestibility coefficients even as protein and energy digestibility improve (Ndelekwute et al., 2018). Because NDF digestibility in poultry depends heavily on microbial fermentation rather than endogenous enzymatic activity, a pH environment that is antimicrobial enough to suppress E. coli and total aerobic counts (as observed in HEBA; Table 4) would be expected to similarly constrain the fiber-fermenting community, even while carbohydrase supplementation continued to support protein and energy release from the same substrate. This would explain why NDF digestibility declined in HEBA despite concurrent improvements in CP and GE digestibility in the same group. Nevertheless, the HEBA maintained protein digestibility at high level, indicating that enzyme–acid supplementation can still support amino acid availability.

The enhancement in digestibility of protein is related to the amino acid profile. Also, hempseed protein has higher level of essential amino acids ratio than soybean. Hemp is rich of arginine and glutamic acid, and good amounts of sulphur-containing amino acids (Shariatmadari, 2023). According to Wang and Xiong (2019), in vitro protein digestibility of hempseed (88 %) is higher than soy protein, and based on bioactive compounds, may improve nutrient absorption.

Higher ileal CP/EE/GE digestibility aligns with reports that xylanase improves nutrient utilization in corn–soy or wheat diets by reducing viscosity and releasing entrapped nutrients (González-Ortiz et al., 2019). β-glucanase depolymerizes soluble β-glucans and shifts carbohydrate disappearance proximally, explaining improved ileal digestibility metrics (Karunaratne et al., 2023).

The breakdown of NSPs and release of encapsulated nutrients are increased by multienzyme supplement. High digesta viscosity due to NSPs in hempseed can diminish by adding carbohydrase blend to feed which lead to degrades cell wall polysaccharides and improving digestibility. Attia et al. (2022) demonstrated similar results regarding the enhancement of nutrient digestibility through enzyme supplementation, particularly in high-fiber diets.

In contrast, DM decreased in the HBA and HEBA groups, which may interact with dietary fiber or modify gut fermentation, leading to reduced DM intake. This could be due to lower gut pH, which shifts the microbial population toward fermenting soluble carbohydrates, leading to a decrease in fiber-degrading bacteria. Although the lower DM digestibility, the HEBA indicating enzyme and acid supplementation still supports amino acid availability, preserved protein digestibility at high. This agrees with findings by Yaseen et al. (2024), who noted that combined additives do not always have additive effects due to complex microbial interactions (Chowdhury et al., 2021).

NDF was significantly lower in the hemp-fed groups (except HE) compared to the control. This suggests that the enzyme can help to mitigate the lower digestibility of hempseed fiber fraction. Then, carbohydrase blend supplementation becomes valuable when dietary fiber increases endogenous losses and reduces nutrient absorption. Complementary acts when enzymes and organic acids are used alongside have been reported; butyrate supports mucosal maturation, while enzymes lower digesta viscosity and release entrapped nutrients (González-Ortiz et al., 2019).

### Blood biochemistry parameters

Dietary treatments significantly affected serum cholesterol, glucose, calcium, phosphorus, globulin concentration, and the albumin-to-globulin (Alb/Glo) ratio, whereas albumin, HDL, LDL, triglycerides, and total protein were not influenced (Table 7). Serum cholesterol concentration was higher in the H group than in the C and HBA groups (P < 0.05), while the HE and HEBA groups showed intermediate values. Serum glucose concentration was lower in the HEBA group than in the C, H, and HBA groups (P < 0.05), whereas the HE group exhibited intermediate values. Serum calcium concentration was greater in the HEBA group than in the control group (P < 0.05), while phosphorus concentration was higher in the HEBA group than in the C and H groups (P < 0.05). Globulin concentration was lower in the HBA group than in the H and HEBA groups (P < 0.05). The albumin-to-globulin ratio was higher in the HBA group than in the H group (P < 0.05).

**Table 7:** Serum biochemical profile of treatments at 28 days of age.

Treatments*	Alb (g/dl)	Chol (mg/dl)	Glu (mg/dl)	HDL (mg/dl)	LDL (mg/dl)	TG (mg/dl)	TP (g/dl)	Ca (mg/dl)	P (mg/dl)	Glo (g/dl)	Alb/Glo
C	2.70	119.3^b^	348.1^a^	40.7	101.8	59.0	4.92	7.87^b^	4.78^b^	1.91^a^^,^^b^	1.43^a^^,^^b^
H	2.68	136.3^a^	342.1^a^^,^^b^	45.0	107.5	63.5	5.53	8.43^a^^,^^b^	5.21^b^	2.24^a^	1.21^b^
HE	2.80	130.4^a^^,^^b^	334.1^b^^,^^c^	41.4	113.4	56.6	5.06	8.84^a^^,^^b^	5.79^a^^,^^b^	1.92^a^^,^^b^	1.52^a^^,^^b^
HBA	3.11	119.0^b^	344.3^a^^,^^b^	41.7	105.5	65.3	5.02	8.22^a^^,^^b^	5.96^a^^,^^b^	1.67^b^	1.88^a^
HEBA	2.68	131.3^a^^,^^b^	330.0^c^	46.9	105.6	57.9	5.77	9.06^a^	6.70^a^	2.21^a^	1.22^a^^,^^b^
SEM	0.207	4.55	2.76	1.76	3.72	2.69	0.335	0.275	0.342	0.103	0.159
*P-value*	0.540	0.042	<0.001	0.089	0.297	0.134	0.323	0.037	0.006	0.003	0.040

a-c Means in the same column with different superscripts are significantly different (p < 0.05).

⁎ Eight replicate cages per treatment. SEM: standard error of the mean. C: Control, H: 16 % hempseed, HE: 16 % hempseed with enzyme, HBA: 16 % hempseed with butyric acid, HEBA: 16 % hempseed with enzyme and butyric acid. Alb: Albumin, Chol: Cholesterol, Glu: Glucose, HDL: High-density lipoprotein, LDL: Low-density protein, TG: Triglyceride, TP: Total protein, Ca: Calcium, P: Phosphorus, Glo: Globulin.

Lack of observation of significant differences in albumin, total protein, HDL, LDL, and triglycerides after hempseed administration was determined and it became clear to us that it does not impair protein metabolism, hepatic function, or lipid transport. Then we can conclude metabolic homeostasis was maintained across treatments.

Nevertheless, the highest level of cholesterol in the H group demonstrates that lipid metabolism alteration can be obtained from hempseed alone, which is probably due to its high fat content and PUFA profile, which can stimulate hepatic lipid synthesis and elevate circulating cholesterol (Montero et al., 2023). Similarly, previous studies (Khan et al., 2010; Sana et al., 2024) are consistent with our observation, while they used hempseed without additives. Counterbalance impact of butyric acid throughout its role in lipid absorption and bile salt metabolism, leading to cholesterol not being raised in the HBA.

Glucose levels were significantly reduced in the HE and HEBA groups, reflecting improved carbohydrate metabolism with multienzyme supplementation (Zhou et al., 2021). Most likely, glucose release elevation, uptake, and lowering the circulating level, induced by starch and NSPs breakdown (Attia et al., 2022). These results also match the observed improvements in FCR for enzyme-fed birds. Mechanistically, this reduction in circulating glucose is consistent with a shift toward more efficient peripheral glucose utilization rather than impaired absorption. Carbohydrase-mediated NSP hydrolysis reduces digesta viscosity and cell-wall encapsulation of starch granules, improving the rate and extent of amylolytic access to starch (Kouzounis et al., 2021); the resulting glucose is absorbed more efficiently and made available for tissue uptake and growth, which would be expected to lower circulating glucose concentrations at a fixed sampling time point rather than reflect reduced carbohydrate digestion. This interpretation is consistent with the concurrent improvement in FCR in the HE and HEBA groups, indicating that glucose was being utilized for growth rather than accumulating in circulation.

Mineral metabolism was notably raised in the HEBA group, which showed the highest calcium and phosphorus concentrations. This is consistent with the dual effect of organic acids (which increase mineral solubility) and carbohydrase (which release minerals bound to phytic acid), as supported by and Anjum and Chaudhry (2010). This is consistent with the established mode of action of organic acids on mineral nutrition: lowering digesta pH increases the ionization and solubility of calcium and phosphorus, while reducing the formation of poorly soluble calcium-phytate complexes that would otherwise limit absorption (El-Saadony et al., 2022). Because carbohydrase supplementation additionally disrupts the hempseed cell-wall matrix in which phytate and bound minerals are physically entrapped, the combination of enzyme and acid in HEBA likely acted on two sequential barriers to mineral availability — chemical solubility and physical entrapment — which is consistent with HEBA showing the highest serum calcium and phosphorus among treatments.

A decrease in the Alb/Glo ratio followed by an increase in globulin occurred in treatments H and HEBA. Globulin elevation reflects humoral immune responses, possibly proving the immunomodulatory effects of hempseed’s bioactive compounds (Sana et al., 2024). This suggests that dietary administration, particularly with a combination of butyric acid and carbohydrase blend, contributes to nutrient metabolism and immune competence.

## Conclusion

The present study demonstrates that incorporating 16 % hempseed as a partial replacement for soybean meal can effectively enhance broiler performance when supported by butyric acid and carbohydrase (xylanase + β-glucanase) supplementation. Apparent ileal digestibility and favorable intestinal morphological adaptations, particularly increased VSA and the CD/VH ratio, indicate improved nutrient absorption and utilization capacity. Protein and energy digestibility, which were initially limited by NSPs, decreased with the addition of carbohydrases, while butyric acid modulated gut microbiota and intestinal pH. Moreover, the combination of carbohydrases and butyric acid improved mineral bioavailability and immune status, as reflected in elevated serum calcium, phosphorus, and globulin levels. Totally, these findings suggest that hempseed, especially when incorporated with carbohydrases blend and butyric acid, enhances nutrient efficiency, intestinal integrity, and physiological stability in broilers, highlighting its potential as a sustainable functional ingredient in poultry nutrition.

## Author contributions

Alireza Shahtalab made substantial contributions to the conduct of the study, including data acquisition, analysis, and interpretation, and prepared the initial draft of the manuscript. He critically reviewed and revised the manuscript for important intellectual content and explicitly approved the exact final version for publication. He agrees to be personally and publicly accountable for all aspects of the work and to ensure that any questions concerning the accuracy or integrity of the research are appropriately investigated and resolved. Farid Shariatmadari made substantial contributions to the conduct of the study, including data acquisition, analysis, and interpretation. He also contributed substantially to the critical review and revision of the manuscript for important intellectual content. He explicitly approved the exact final version for publication and agrees to be personally and publicly accountable for all aspects of the work, including ensuring that any questions concerning its accuracy or integrity are appropriately investigated and resolved. Mohammad Amir Karimi Torshizi made substantial contributions to the conduct of the study, including data acquisition, analysis, and interpretation. He critically reviewed and revised the manuscript for important intellectual content. He explicitly approved the exact final version for publication and agrees to be personally and publicly accountable for all aspects of the work, including ensuring that any questions concerning its accuracy or integrity are appropriately investigated and resolved. Hamed Ahmadi made substantial contributions to the conduct of the study, including data acquisition, analysis, and interpretation. He also contributed substantially to the critical review, revision, and editing of the manuscript for important intellectual content. He explicitly approved the exact final version for publication and agrees to be personally and publicly accountable for all aspects of the work, including ensuring that any questions concerning its accuracy or integrity are appropriately investigated and resolved. All authors confirm that they meet all four of the journal’s authorship requirements and accept full responsibility and accountability for their respective contributions and for the integrity of the published work.

## Disclosures

The authors declare that they have no known competing financial interests or personal relationships that could have appeared to influence the work reported in this paper.
